# Barbed versus traditional sutures for wound closure in knee arthroplasty: a systematic review and meta-analysis

**DOI:** 10.1038/srep19764

**Published:** 2016-01-25

**Authors:** Wei Zhang, Deting Xue, Houfa Yin, Hui Xie, Honghai Ma, Erman Chen, Dongcai Hu, Zhijun Pan

**Affiliations:** 1 Department of Orthopedics, Second Affiliated Hospital, School of Medicine, Zhejiang University, 310009, Hangzhou, People’s Republic of China; 2 Eye Center, Second Affiliated Hospital of Zhejiang University School of Medicine, Hangzhou, Zhejiang Province, China; 3 Department of Thoracic surgery, First Affiliated Hospital of Zhejiang University School of Medicine, Hangzhou, Zhejiang Province, People’s Republic of China

## Abstract

Sutures are an increasing focus of research in knee arthroplasty (KA). Whether knotless barbed sutures (KBS) are safe and efficient in KA remains controversial. The objective of our study is to compare the clinical outcomes of KA according to wound closure method: KBS versus knotted traditional sutures (KTS). To clarify this, we conducted a systematic review and meta-analysis. Nine articles involving 10 studies were included in this study. The dataset consisted of 1729 patients with 1754 KA. Among these, 814 patients’ wounds were closed with KBS and 915 with KTS. Our analysis indicates that KBS is preferable for KA wound closure given its shorter wound closure time and lower total cost; postoperative Knee Society scores and complication rates were similar to those of surgeries using KTS. The subgroup analysis revealed that closure of arthrotomy with KBS appears to be associated with a lower risk of complications. This meta-analysis indicates that use of KBS in KA reduces operative time and cost. KBS is the preferred option for wound closures, including arthrotomy and reattachment of subcutaneous and subcuticular tissues. Given the possible biases, adequately powered and better-designed studies with longer follow-up are required to reach a firmer conclusion.

As the population ages and medical technology improves, the rate of knee arthroplasties (KA) has increased considerably over the past two decades. According to a recent survey, the rate of KA increased by 59.4% from 1991 to 2010: that is, from 3.2 to 5.1 per 10,000 people^1^. This rapid growth in the number of surgeries has also coincided with innovation in surgical procedures, minimizing complications and improving postoperative function. Moreover, improper soft tissue handling remains a risk factor for complications after KA. Thus, sutures are an increasing focus of research in this field, as their quality is crucial to minimize wound complications and withstand forces across the incision during early postoperative knee motion^2^.

The knotless barbed suture (KBS) was first described by R.A. Mckenzie in 1967^3^, and has since been adopted in several surgical fields^4^. KBS has been demonstrated to provide shorter closure time and better soft-tissue repair than knotted traditional suture (KTS) in plastic surgery, urology and obstetrics^5^,^6^,^7^. However, whether KBS is safe and efficient in KA remains controversial. Several studies have found that KBS provides several advantages, including elimination of the need for knot tying and handling of multiple sutures, shorter closure time, use of less suture material and improved tissue distribution^8^,^9^,^10^. Moreover, postoperative clinical outcomes are similar to those of surgeries using KTS^2^,^4^,^11^,^12^,^13^,^14^. In contrast, Campbell *et al*. found that KBS use is associated with a higher rate of infections requiring antibiotics than wound closure with KTS^15^. Furthermore, work by Smith *et al*. suggests that KBS is associated with greater frequency and severity of wound-related complications^16^.

Therefore, this quantitative meta-analysis was undertaken to inform clinical practice regarding which of the two suture methods (KBS or KTS) leads to better outcomes and lower rates of complications in KA.

## Materials and Methods

This meta-analysis was performed strictly according to the guidelines for ‘preferred reporting items for systematic reviews and meta-analyses’ (the ‘PRISMA’ statement)^17^.

### Data retrieval

Two independent researchers searched PubMed, Embase, and the Cochrane Library. Data were last updated on March 4, 2015. The following keywords or corresponding Medical Subject Headings (MeSH) were used: “barbed” or “knotless” and “knee arthroplasty” or “knee replacement” or “joint replacement” or “joint arthroplasty”. Reference lists of the relevant articles were also reviewed for additional relevant studies. The search was not limited by language.

### Inclusion criteria

Studies were identified according to the following inclusion criteria: 1) participants: human with relevant diseases requiring surgical intervention, 2) intervention: primary KA, 3) comparison: wound closure with the use of KBS (closing at least subcutaneous and subcuticular tissue) versus KTS (closing subcutaneous and subcuticular tissue as well as the arthrotomy), 4) Outcomes: at least one of the following: wound closure time, complications, cost, postoperative function, 5) Methodological criterion: a prospective study, a case-control study or a cohort study.

The following exclusion criteria were used: 1) insufficient data to estimate an odds ratio (OR) or weighted mean difference (WMD), 2) non-human or cadaver subjects, 3) redundant publications, 4) non-primary research (editorials, commentaries, etc.).

### Data extraction

Two authors extracted relevant data independently, including the first author’s name, study design, publication year, number of patients in each group, average patient age, gender ratio, details on the suture method and technique, total complications and major complications, total cost of wound closure, and postoperative Knee Society scores (KSS). Major complications were defined as those requiring further surgical interventions. Total cost of wound closure included both suture material and operating room time. The cost of operating room time estimates are based upon the average cost for professional staff and resources required for these cases. Intention-to-treat (ITT) data were used when available. Data in other forms, such as medians, interquartile ranges, and means ± 95% confidence interval (CI), were converted to means ± SD following the guidelines in the Cochrane Handbook for Systematic Reviews of Interventions 5.0.2.

### Quality assessment

Using a 12-item scale^18^, the methodological quality of each included study was assessed by two independent researchers. The 12-item scale consisted of the following: adequate randomization, concealment of allocation, patient blinding, care provider blinding, outcome assessor blinding, dropout rate, ITT analysis, avoidance of selective reporting, similarity of baseline characteristics, similarity or absence of cofactors, patient compliance, and similarity of timing. Disagreements were evaluated by kappa test and consensus was achieved by discussion with the corresponding author.

### Statistical analysis

Statistical analyses were performed using Stata software (ver. 12.0; StataCorp LP, College Station, TX, USA). Weighted mean differences (WMD) with 95% confidence interval (95% CI) were calculated for continuous data and odds ratios (OR) with 95% CIs were calculated for dichotomous data. Statistical heterogeneity was assessed by Q-test and *I*^2^. *I*^2^ values of 25%, 50%, and 75% were considered to indicate low, moderate, and high heterogeneity, respectively^19^. If *P* > 0.1 and *I*^2^ < 50%, a fixed-effects model was used; otherwise, a random-effects model was used. For substantial heterogeneity (*I*^2^ > 50%), a sensitivity analysis (backward elimination step-wise regression analysis) was conducted by omitting one study sequentially to examine the influence of each.

The stratified subgroup analysis compared outcomes according to suture type (within the KBS group, whether the arthrotomy was closed with KBS; in the KTS group, whether subcuticular tissue was closed with a running or an interrupted suture) or study design (randomized controlled trials (RCTs) versus non-RCTs).

Publication bias was assessed by Egger’s test and Begg’s test. For all statistical analyses, with the exception of heterogeneity, a value of *P* < 0.05 was considered to indicate statistical significance, and all tests were two-sided.

## Results

### Study selection

**Figure 1:**
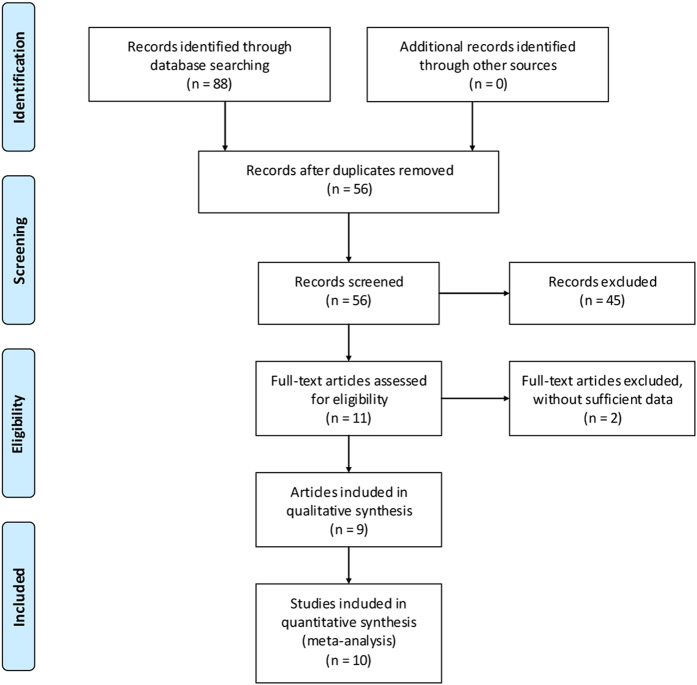
Flow chart summarizing the selection process of studies (From: Moher D, Liberati A, Tetzlaff J, Altman DG, The PRISMA Group (2009). Preferred Reporting Items for Systematic Reviews and MetaAnalyses: The PRISMA Statement. PLoS Med 6(6): e1000097. doi: 10.1371/journal.pmed1000097).

The article selection process is shown in Fig. 1. The search yielded 88 potentially relevant articles: 46 from PubMed, 32 from Embase, and 10 from the Cochrane library. Of these, 32 duplicates were removed using Endnote software. Upon review of titles and abstracts of the 56 remaining articles, the full text of 11 articles was retrieved. Because sufficient data were not available in two articles, they were excluded^20^,^21^, leaving a total of nine articles included in this study^2^,^4^,^11^,^12^,^13^,^14^,^15^,^16^.

### Study characteristics

The characteristics of the nine articles are presented in Table 1. These 9 articles, published between 2010 and 2015, actually include 10 studies; that by Smith *et al*.^16^ included a RCT and a retrospective study. Of these, four were RCTs, one was a prospective cohort study, and five were retrospective studies. The dataset consisted of 1729 patients, including 1754 KA. Among these, 814 patients’ wounds were closed with KBS and 915 with KTS. Each study included between 18 and 416 patients. The average age, gender ratio, and surgical site were also noted. In each study, the demographic characteristics of the two groups were similar.

For KA, details on the exact type of suture used and the method of placement are in Table 2. For surgeries involving both of KBS and KTS, placement and type of stitches varied. Among surgeries using the KBS method, the arthrotomy was closed with an interrupted knotted suture in two studies^15^,^22^, while others used a running KBS. In the KTS group, subcuticular tissue was closed with a running suture in three studies, while others used the interrupted suture technique.

### Study quality

Table 3 shows the quality of the included studies. Of these, only one study was high quality; the others were of moderate quality. There was excellent inter-rater agreement between the investigators regarding eligibility (κ = 0.78).

### Meta-analysis results

#### Wound closure time

In KA using the KBS method, wound closure times were on average 3.56 minutes shorter than in those using KTS (*n* = 971, WMD = −3.56, 95% CI = −5.05 to −2.08, *P* < 0.01, *I*^2^ = 94%, *P* < 0.01). We could not eliminate heterogeneity through a sensitivity analysis, and thus a random-effects model was used.

#### Risk of total complications

No significant difference was detected in total complication rate between the two groups (*n* = 1729, OR = 0.98, 95% CI = 0.51 to 1.87, *P* = 0.95, *I*^2^ = 56%, *P* = 0.02). Heterogeneity was moderate in the pooled result (*I*^2^ = 56%); a sensitivity analysis was thus performed. Exclusion of data from the Campbell *et al*. study^12^ decreased heterogeneity significantly from 56% to 6%. However, there was still no difference in total risk of complications between the two groups (*n* = 1313, OR = 0.73, 95% CI = 0.46 to 1.15, *P* = 0.17, *I*^2^ = 6%, *P* = 0.38).

#### Major complications and other complications

Patients in both groups experienced similar rates of major complications (*n* = 1634, OR = 1.17, 95% CI = 0.67 to 2.18, *P* = 0.62, *I*^2^ = 17%, *P* = 0.30).

Full details of complications are summarized in Table 4. There were no differences between the two groups in rates of superficial infection, deep infection, wound dehiscence, arthrofibrosis, hematoma, or suture abscess.

#### Postoperative KSS

Three studies examined postoperative function in terms of KSS at 6 weeks after surgery. KA patients receiving KBS had a postoperative KSS value 0.98 points greater than those receiving KTS (*n* = 677, WMD = 0.98, 95% CI = 0.69 to 1.26, *P* < 0.01, *I*^2^ = 31%, *P* = 0.24).

#### Cost

**Figure 2:**
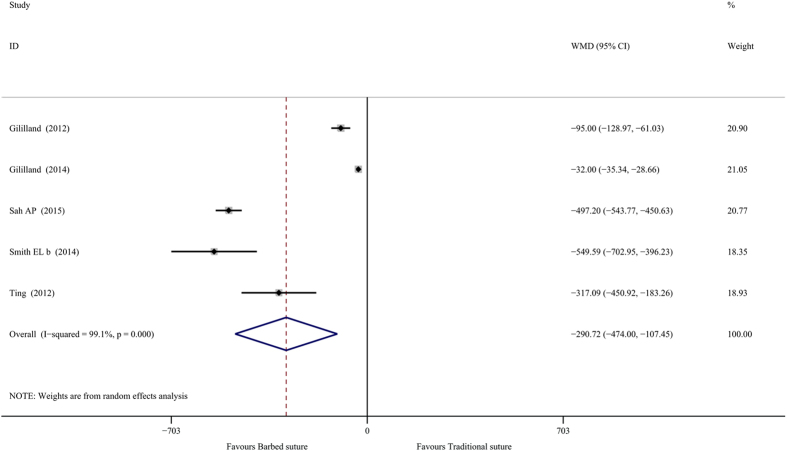
Forest plot for total costs analysis.

The cost differences in terms of material between the two groups were summarized in Table 5. Upon analysis of the pooled cost data, KBS was associated with 290.72 USD lower costs than KTS (*n* = 871, WMD = −290.72, 95% CI = −474.00 to −107.45, *P* = 0.002, *I*^2^ = 99.1%, *P* < 0.01, Fig. 2).

### Result of the subgroup analysis

Table 6 shows the results of the subgroup analysis. No significant difference was observed in risk of complications according to different types of study design (Supplement materials 1–3). Using KBS to close arthrotomies appears to lead to a lower total risk of complications relative to KTS. However, among surgeries employing KBS, rates of all complications, major complications (deep infection and others complications which required further surgical interventions, including polyethylene exchange, irrigation and debridement), and superficial infection were greater if KBS was not used for arthrotomy closure. Other factors did not differ significantly between the two groups.

### Publication bias

Begg’s test (*P* = 0.89, continuity corrected) and Egger’s test (*P* = 0.108) indicated that publication bias did not affect our results.

## Discussion

Our study found that in KA, KBS was associated with shorter wound closure times and lower costs than KTS. Meanwhile, closure of arthrotomies with KBS led to similar postoperative function and lower risk of all complications within the KBS group.

KBS was associated with a shorter time to wound closure in our study, confirming results of previous studies^20^,^21^,^23^,^24^. Stephens *et al*. found that KBS saved approximately 4 minutes in KA compared with KTS^21^. Moreover, in a study by Mansour *et al*. of spinal fusions, KBS resulted in a 40% reduction in wound closure time^23^. KBS is self-anchoring, requiring no knots, thus allowing faster closure^13^. However, we observed substantial heterogeneity in wound closure time, likely resulting from variation in KBS and KTS technique (Table 2). Among included studies, surgical approaches employing KBS varied with regard to suture method for arthrotomy and superficial skin closure, making significant heterogeneity in wound closure time unavoidable.

We observed no difference in complication rate between surgeries employing KBS versus KTS, consistent with previous studies^20^,^21^. Theoretically, knots may place uneven pressure on soft tissue, resulting in ischemia, while adsorption of bulky knots may cause local tissue inflammation and scarring and serve as a potential nidus for infection. Moreover, KBS provides more uniform tissue tension to reduce local ischemia, thereby decreasing risk of wound complications^12^. However, KBS is a running suture, which can strangulate the vascular supply and inhibit soft tissue healing^16^. Additionally, Shermak *et al*. found that KBS increases risk of wound healing complications in the arm^25^, which they speculated results from increased surface area caused by barbs and continuous suturing, promoting spread of inflammation along the length of the closure^25^.

Moreover, we also found that closure of arthrotomy, subcutaneous, and subcuticular tissues with KBS appeared to decrease the total complication rate in KA. Conversely, arthrotomy closure by other methods in KBS group appeared to lead to a higher complication rate. A cadaver study simulating tense hemarthrosis found 74% lower leakage from a barbed suture arthrotomy closure than from KTS^9^. Arthrotomy leakage was minimal when distal arthrotomy closure was tight. Moreover, a biomechanical study found that KBS arthrotomy closure provides similar performance to interrupted KTS upon cyclical loading. Furthermore, KBS performed better than interrupted sutures when the repair was intentionally damaged^8^. Thus, using KBS to close arthrotomy is very important. Whereby achieving a more watertight wound closure may limit the risk of complications^9^.

At early stages (<6 weeks), patients receiving KBS had 0.98 higher postoperative KSS than those receiving KTSs in our study; this difference was not clinically significant. Likewise, Sah *et al*. found that range of knee motion is similar for both suture methods for up to one year^2^. An adequately powered RCT with long-term follow-up is necessary to determine the effect of suture method on recovery of function.

Our analysis found that KBS to significant cost savings. Similarly, Mansour *et al*. found that KBS closure of spinal fusion incisions resulted in hospital charges for operation time that were 884.60 USD lower than those of surgeries using KTS^23^. The cost of operating room time estimates are based upon the average cost for professional staff and resources required for these cases. The average cost of operating room was USD 62 per minute (range, USD 22–133/minute) in 100 United States hospitals^26^. In our study, KBS were associated with a mean savings of 3.56 minutes, which is in the range of 2.08 to 5.05 minutes with use of this suture device. Of note, the material cost with KTS could be saved USD 91.93, at most, in these included studies. Though barbed closure materials are more expensive than those for KTS, shorter surgery time leads to a reduced total cost.

To our knowledge, this is the first meta-analysis comparing the risk of complications and postoperative function between KBS and KTS for KA that includes all available comparative evidence and comprehensively investigates differences in the clinical outcomes. However, it has the following limitations. Most importantly, surgeries classified as KBS and KTS included multiple methods for closing superficial skin (Table 2), making significant heterogeneity in wound closure time and total cost unavoidable. Moreover, there is a lack of uniform technique of KTS. Second, as few RCTs in this area have been performed (perhaps because KBS remains relatively new in KA), our study included several non-RCTs, which inevitably involved recall and interviewer bias, which likely weakened our analysis. Nonetheless, excluding non-RCTs would have underpowered the analysis, increase the risk of false-negative errors and influence the accuracy of our findings. Demographic characteristics were similar between the two groups in all included studies, suggesting that selection bias was limited. Third, follow-up duration was relatively short, preventing examination of long-term outcomes, especially postoperative function. Furthermore, the bias might also be produced by the variations of stitches, such as V-Loc stitch and Quill stitch in KBS.

## Conclusions

Based on available evidence, use of KBS in KA leads to shorter operation times and lower total costs. Closure of arthrotomy and subcutaneous and subcuticular tissues by KBS yields similar postoperative function and lower total complication risk when compared with KTS. We thus conclude that KBS is an optimal approach for closure of arthrotomies and subcutaneous and subcuticular tissues in KA. Given the relevant possible biases in our study, adequately powered and better-designed studies with long-term follow-up are required to reach a firmer conclusion.

## Additional Information

**How to cite this article**: Zhang, W. *et al*. Barbed versus traditional sutures for wound closure in knee arthroplasty: a systematic review and meta-analysis. *Sci. Rep*. **6**, 19764; doi: 10.1038/srep19764 (2016).

## Supplementary Material

Supplementary Information

## Figures and Tables

**Table 1:** Study characteristics.

First author	Publication year	Design	Group size (patients)	Average age (Years)	Gender ratio (Male/Female)		BMI(Kg/m^2^)		Knees	Clinical outcomes	Follow-up Periods (Months)
			Barbed/Traditional	Barbed/ Traditional	Barbed	Traditional	Barbed	Traditional			
Campbell^15^	2014	Prospective Cohort study	169/247	65.3/67.6	37/132	62/185	NA	NA	416	Complications	12
Eickmann^11^	2010	Retrospective study	90/88	67.6/68.0	32/54	23/56	NA	NA	178	Complications	3
Gililland^12^	2014	Multicenter RCT	191/203	64/63	77/144	77/126	33	33	411	Closure time	1.5
										Complications	
										KSS	
										Total closure costs	

Gililland^13^	2012	Retrospective study	98/85	61/63	32/66	30/55	32	33	191	Closure time	1.5
										Total closure costs	
										Complications	
										KSS	
Maheshwari^4^	2014	Retrospective study	115/75	65/61	22/93	16/59	34	34	190	Closure time	6
										Complications	
Patel^22^	2012	Retrospective study	23/130	NA	NA	NA	NA	NA	153	Complications	NA
Sah AP^2^	2015	Single-center RCT	50/50	68/68	21/29	21/29	30	30	100	Closure time	12
										Complications	
										KSS, ROM	
										Total closure costs	
Smith a^16^	2014	Single-center RCT	10/8	59/64	9/9	6/10	33	30	18	Closure time	NA
										Complications	
										Total costs	
Smith b^16^	2014	Respective study	51/11	NA	NA	NA	NA	NA	62	Complications	NA
Ting^14^	2012	RCT	17/18	64/63	8/23	8/21	31	32	35	Closure time	3
										Complications	
										Total closure costs	
Total	–	–	814/915	–	–	–			1754	–	–

NA: not available.

**Table 2:** Details of the suture type and method of placement for knee arthroplasty.

Author	Publication year	Suture method compared	
		Barbed	Traditional
Campbell^15^	2014	Arthrotomy: Interrupted #1 Maxon polyglyconate	Arthrotomy: Interrupted #1 Maxon polyglyconate
		Subcutaneous: Running 2.0 V-Loc	Subcutaneous:Interrupted 2.0 Vicryl
		Skin: Running 3.0 V-Loc	Skin: Staples
Eickmann^11^	2010	Arthrotomy: Running #2 Quill	Arthrotomy: Interrupted 1-0 Vicryl
		Subcutaneous tissue: Running #2Quill	Subcutaneous: Interrupted 2-0 Vicryl
		Subcuticular: Running #2Quill	Subcuticular: Running 4-0 Monocryl
		Skin: Tissue adhesive	Skin: Tissue adhesive
Gililland^12^	2014	Arthrotomy: Running #2 Quill	Arthrotomy: Interrupted #1 Ethibond
		Subdermal: Running #0 Quill	Subdermal: Interrupted 2-0 Monocryl
		Skin: Staples	Skin: Staples
Gililland^13^	2012	Arthrotomy: Running #2 Quill	Arthrotomy: Interrupted #1 Ethibond
		Subdermal: Running #0 Quill	Subdermal: Interrupted 2-0 Monocryl
		Skin: Staples	Skin: Staples
Maheshwari^4^	2014	Arthrodtomy: Interruptted#1 Ethibond and Running 2# Quill	Arthrodtomy: Interrupted # 1Ethibond/ 1-0 Vicryl
		Subcutaneous: Running 0# Quill	Subcutaneous: Interrupted 0-Vicryl
		Skin: Staples	Skin:Interrupted 3-0 Ethicon
Patel RM^22^	2012	Arthrotomy: Interrupted 1-0 Vicryl	Arthrotomy: Interrupted 1-0 Vicryl
		Subcutaneous: Running 3.0 V-Loc	Subcutaneous: Interrupted 2-0 Vicryl
		Subcuticular: Running 3.0 V-Loc	Subcuticular: Interrupted 3.0 Biosyn
		Skin: Staples	Skin: Staples
Sah AP^2^	2015	Arthrodtomy: Running 2-0 Quill	Arthrodtomy: Interrupted 2-0 Vicryl
		Subcutanoueous: Running 2-0 Quill	Subcutanoueous: Running 2-0 Monocryl
		Subcuticular: Running 2-0 Quill	Subcuticular: Running 3-0 Monocryl
		Skin: Unclear	Skin: Unclear
Smith EL^16^	2014	Arthrotomy: Running #2 Quill	Arthrotomy: Interrupted #1 Ethibond
		Subcutaneous: Running #0 Quill	Subcutaneous: Interrupted 2.0 Vicryl
		Subcuticular: Running 2-0 Quill	Subcuticular: Running 3-0 Monocryl
		Skin: Unclear	Skin: Unclear
Ting^14^	2012	Arthrotomy: Running #2 Quill	Arthrotomy: Interrupted 1-0 Vicryl
		Subcutaneous: Running #0 Quill	Subcutaneous: Interrupted 2.0 Vicryl
		Subcuticular: Running 2-0 Quill	Subcuticular: Interrupted 2-0 Monocryl
		Skin: Adhesive and staples	Skin: Adhesive and staples

V-Loc and Quill are the absorbable barbed materials for suture.

**Table 3:** Study quality.

First author	Randomized adequately^1^	Allocation concealed	Patient blinded	Care provider blinded	Outcome assessor blinded	Acceptable drop-out rate^2^	ITT analysis^3^	Avoided selective reporting	Similar baseline	Similar or avoided cofactor	Patient compliance	Similar timing	Quality^4^
Campbell^15^	No	No	No	No	No	Yes	Yes	Unclear	Yes	Yes	Yes	Yes	Moderate
Eickmann^11^	No	No	No	No	No	Yes	Yes	Unclear	Yes	Yes	Yes	Yes	Moderate
Gililland^12^	Yes	Unclear	Yes	No	Unclear	Yes	Yes	Unclear	Yes	Yes	Yes	Yes	Moderate
Gililland^13^	No	No	No	No	No	Yes	Yes	Unclear	Yes	Yes	Yes	Yes	Moderate
Maheshwari^4^	No	No	No	No	No	Yes	Yes	Unclear	Yes	Yes	Yes	Yes	Moderate
Patel^22^	No	No	No	No	No	Yes	Yes	Unclear	Yes	Yes	Yes	Yes	Moderate
Sah AP^2^	Unclear	Unclear	Unclear	No	Unclear	Yes	Yes	Unclear	Yes	Yes	Yes	Yes	Moderate
Smith a^16^	Unclear	Yes	Unclear	No	No	Yes	Yes	Unclear	Yes	Yes	Yes	Yes	Moderate
Smith b^16^	No	No	No	No	No	Yes	Yes	Unclear	Yes	Yes	Yes	Yes	Moderate
Ting^14^	Yes	Yes	Unclear	No	Unclear	Yes	Yes	Unclear	Yes	Yes	Yes	Yes	High

^1^Only if the method of sequence made was explicitly introduced could get a ‘Yes’.

^2^Drop-out rate < 20% could get a ‘Yes’, otherwise ‘No’.

^3^ITT = intention-to-treat, only if all randomized participants were analyzed in the group they were allocated to could receive a ‘Yes’.

^4^“Yes” items more than 7 means ‘High’; more than 4 but no more than 7 means ‘Moderate’; no more than 4 means ‘Low’.

**Table 4:** The comparison in complications between barbed suture and traditional suture.

Complications	No. of studies	Adverse Events Rate		OR [CI]	I^2^	*P*value
		Barbed	Traditional			
Overall complications	10	70/814	70/915	0.98 [0.51, 1.87]	56%	0.95
Major complications^1^	8	21/754	20/880	1.17 [0.63, 2.18]	17%	0.62
Superficial Infection	6	40/703	40/846	1.46 [0.92, 2.31]	50%	0.11
Deep Infection	2	9/43	4/29	2.13 [0.20, 22.26]	63%	0.63
Wound Dehiscence	7	18/695	12/916	1.45 [0.67, 3.10]	0%	0.16
Arthrofibrosis	4	13/388	15/449	1.09 [0.52, 2.29]	0%	0.81
Hematoma	2	2/188	5/173	0.36 [0.07, 1.87]	0%	0.22
Suture abscess	3	15/410	14/500	1.35 [0.66, 2.79]	36%	0.41

^1^Major complications defined as those requiring further surgical interventions.

**Table 5:** Relevant cost and closure time in barbed and traditional groups.

First Author	Publication year	The cost of operating room time (per minute)	Average suture material costs		Total closure time in operating room (minute)		Total cost for suture (Mean (SD/range))	
			Barbed	Traditional	Barbed	Traditional	Barbed	Traditional
Campbell^15^	2014	NA	NA	NA	NA	NA	NA	NA
Eickmann^11^	2010	NA	NA	NA	NA	NA	NA	NA
Gililland^12^	2014	$28	$24	$2	9.8 (4.22)	14.4 (3.98)	$324 (118)	$419 (116)
Gililland^13^	2012	$28	$43	$6	19.6 (18.5–20.7)	22.0 (20.7–23.3)	$595 (564–626)	$627 (590–663)
Maheshwari^4^	2014	NA	$66.78	$82.59	31	30	NA	NA
Patel RM^22^	2012	NA	NA	NA	NA	NA	NA	NA
Sah AP^2^	2015	$48	$82	$32	11.4 (2.2)	16.1 (2.1)	$307.6 (134.4)	$804.8 (100.8)
Smith EL^16^	2014	$66	$106.33	$14.40	16.78 (3.28)	26.50 (6.83)	$1213.8 (216.48)	$1763.4 (450.78)
Ting^14^	2012	$103	$52.84	$9.43	9.2 (1.875)	12.7 (3.075)	$1000.44 (193.125)	$1317.53 (316.725)

NA, not available.

**Table 6:** Subgroup analysis of the included studies between KBS and KTS based on influential factors.

Factors	Total complications				Major complications				Superficial Infection				Wound dehiscence			
	subgroup	OR (95% CI)	P value	I^2^	subgroup	OR (95% CI)	P value	I^2^	subgroup	OR (95% CI)	P value	I^2^	subgroup	OR (95% CI)	P value	I^2^
Design	RCT (4)	0.98 [0.33, 2.92]	0.26	49%	RCT (2)	1.06 [0.18, 6.24]	0.61	34%	–	–	–	–	RCT (2)	0.34 [0.03, 3.30]	0.56	0%
	Non-RCT (6)	0.94 [0.38, 2.29]	0.68	64%	Non-RCT (6)	1.19 [0.61, 2.31], 4.31]	0.95	0%	–	–	–	–	Non-RCT (5)	1.85 [0.79, 4.31]	0.35	0%
Arthrotomy with KBS in knee arthroplasty	Yes(5)	0.58 [0.35, 0.98]	0.04	4%	Yes(4)	0.55 [0.23, 1.29]	0.17	0%	Yes(4)	0.75 [0.38, 1.49]	0.41	0%	Yes(5)	0.55 [0.16, 1.90]	0.34	0%
	**No(2)**	**3.05 [1.75, 5.30]**	**<0.01**	**0%**	**No(2)**	**4.60 [1.34, 15.81]**	**0.02**	**0%**	**No(2)**	**3.85 [1.71, 8.67]**	**<0.01**	**0%**	No(2)	3.24 [0.93, 11.24]	0.06	0%
Subcuticular tissue with a running suture in knee arthroplasty	Yes(6)	1.27 [0.59, 2.69]	0.54	69%	Yes(6)	1.24 [0.63, 2.45]	0.53	36%	–	–	–	–	Yes(4)	2.19 [0.80, 5.96]	0.13	0%
	No(4)	0.78 [0.36, 1.69]	0.63	19%	No(2)	0.78 [0.17, 3.55]	0.75	0%	–	–	–	–	No(4)	0.74 [0.25, 2.18]	0.58	0%
