# Supplementary material for: Barbed versus traditional sutures for wound closure in knee arthroplasty: a systematic review and meta-analysis
Source: Sci Rep. 2016 Jan 25;6:19764. doi: 10.1038/srep19764 (PMC4726393; doi:10.1038/srep19764)
Supplement: Supplementary Information [file srep19764-s1.doc]

**Title:** Barbed versus traditional sutures for wound closure in joint arthroplasty: a systematic review and meta-analysis

**Author names:** Wei Zhang, MD^1^, Deting Xue, MD^1^, Houfa Yin, MD^2^, Hui Xie, MD^1^, Honghai Ma, MD^3^, Erman Chen, MD^1^, Dongcai Hu, MD^1^, Zhijun Pan, MD^1^

**Supplement material 1:** Subgroup analysis for overall complications between randomized controlled trials and non-randomized controlled trials


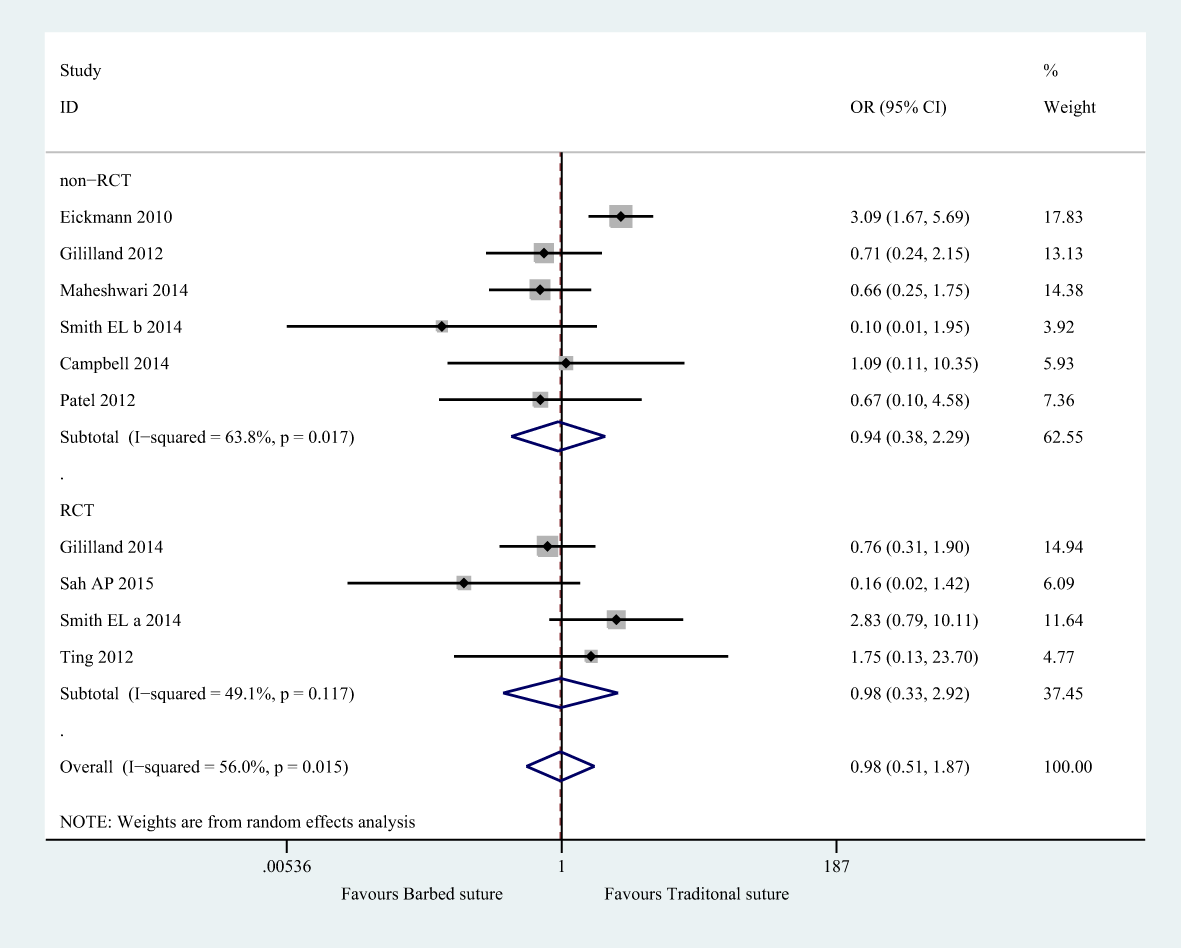


**Supplement material 2:** Subgroup analysis for major complications between randomized controlled trials and non-randomized controlled trials


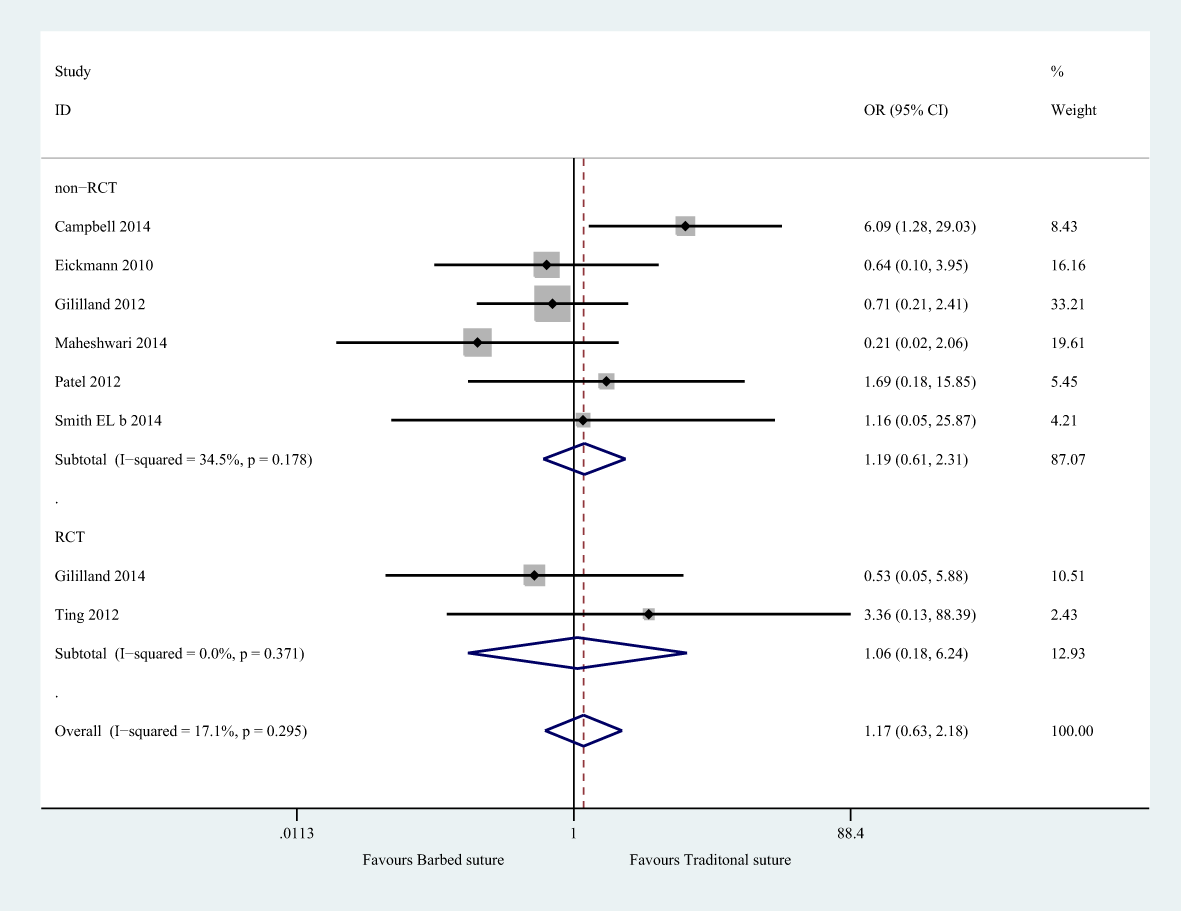


**Supplement material 3:** Subgroup analysis for wound dehiscence between randomized controlled trials and non-randomized controlled trials

**
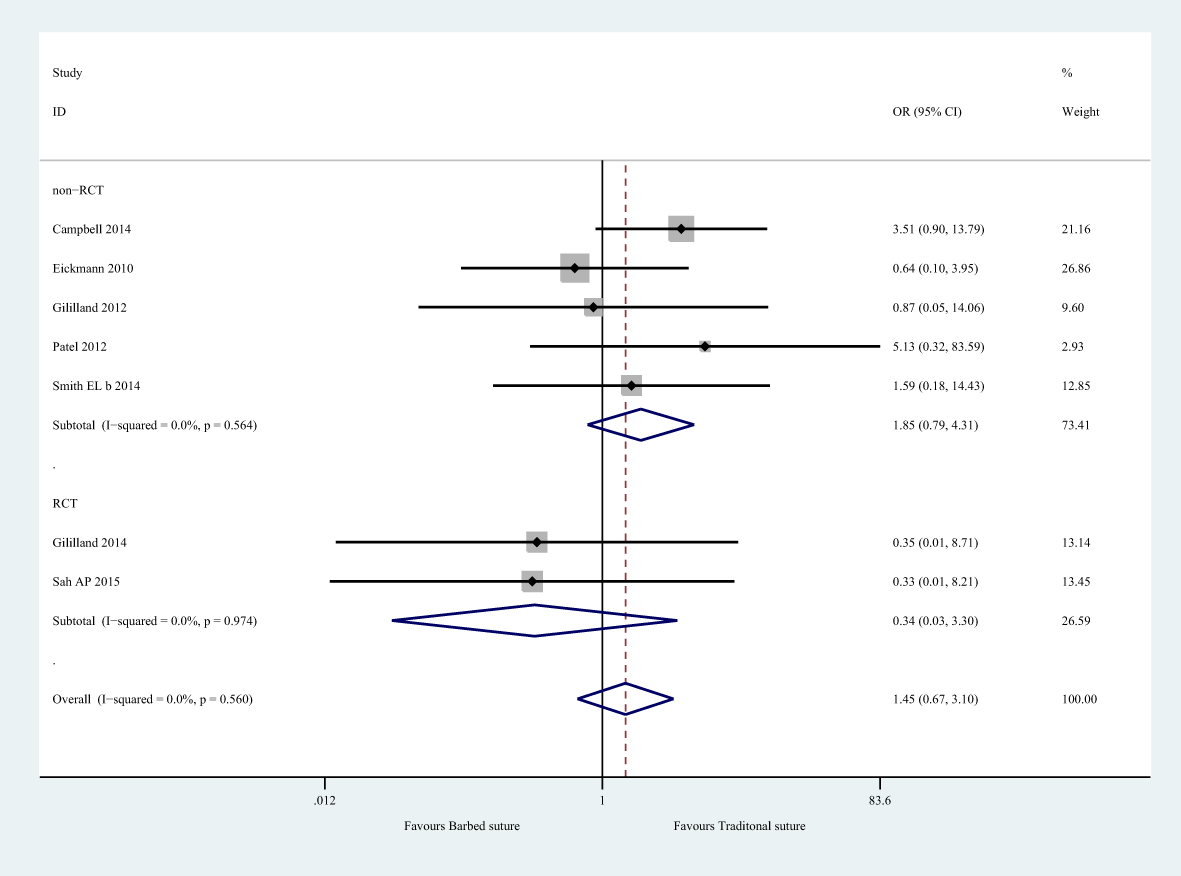
**
